# Recent Transmission Clustering of HIV-1 C and CRF17_BF Strains Characterized by NNRTI-Related Mutations among Newly Diagnosed Men in Central Italy

**DOI:** 10.1371/journal.pone.0135325

**Published:** 2015-08-13

**Authors:** Lavinia Fabeni, Claudia Alteri, Nicoletta Orchi, Caterina Gori, Ada Bertoli, Federica Forbici, Francesco Montella, Alfredo Pennica, Gabriella De Carli, Massimo Giuliani, Fabio Continenza, Carmela Pinnetti, Emanuele Nicastri, Francesca Ceccherini-Silberstein, Claudio Maria Mastroianni, Enrico Girardi, Massimo Andreoni, Andrea Antinori, Maria Mercedes Santoro, Carlo Federico Perno

**Affiliations:** 1 National Institute for Infectious Diseases L. Spallanzani—IRCCS, Rome, Italy; 2 University of Rome Tor Vergata, Rome, Italy; 3 University Hospital Tor Vergata, Rome, Italy; 4 S. Giovanni Addolorata Hospital, Division of Clinical Immunology, Rome, Italy; 5 S.Andrea Hospital, Sapienza University of Rome, Rome, Italy; 6 IRCSS San Gallicano, Rome, Italy; 7 Infectious Diseases Unit, Sapienza University, Polo Pontino, Latina, Italy; University of Pittsburgh, UNITED STATES

## Abstract

**Background:**

Increased evidence of relevant HIV-1 epidemic transmission in European countries is being reported, with an increased circulation of non-B-subtypes. Here, we present two recent HIV-1 non-B transmission clusters characterized by NNRTI-related amino-acidic mutations among newly diagnosed HIV-1 infected men, living in Rome (Central-Italy).

**Methods:**

Pol and V3 sequences were available at the time of diagnosis for all individuals. Maximum-Likelihood and Bayesian phylogenetic-trees with bootstrap and Bayesian-probability supports defined transmission-clusters. HIV-1 drug-resistance and V3-tropism were also evaluated.

**Results:**

Among 534 new HIV-1 non-B cases, diagnosed from 2011 to 2014, in Central-Italy, 35 carried virus gathering in two distinct clusters, including 27 HIV-1 C and 8 CRF17_BF subtypes, respectively. Both clusters were centralized in Rome, and their origin was estimated to have been after 2007. All individuals within both clusters were males and 37.1% of them had been recently-infected. While C-cluster was entirely composed by Italian men-who-have-sex-with-men, with a median-age of 34 years (IQR:30–39), individuals in CRF17_BF-cluster were older, with a median-age of 51 years (IQR:48–59) and almost all reported sexual-contacts with men and women. All carried R5-tropic viruses, with evidence of atypical or resistance amino-acidic mutations related to NNRTI-drugs (K103Q in C-cluster, and K101E+E138K in CRF17_BF-cluster).

**Conclusions:**

These two epidemiological clusters provided evidence of a strong and recent circulation of C and CRF17_BF strains in central Italy, characterized by NNRTI-related mutations among men engaging in high-risk behaviours. These findings underline the role of molecular epidemiology in identifying groups at increased risk of HIV-1 transmission, and in enhancing additional prevention efforts.

## Introduction

In the last five years, more than 100,000 new cases of HIV-1 infection have been reported in European countries [1]. A considerable proportion of these new diagnoses were involved in well-defined clusters (24–34% of the overall), prevalently characterised by high-risk sexual behaviours [2,3].

Clustering of transmitted drug-resistance has also been frequently reported across European and North-American countries [4,5], including cases involving recent infections. Thus, despite the control strategies, primary/recent infection remains a critical period for onward transmission of HIV [6].

In the past decade, an increase of non-B subtypes and circulating recombinant forms (CRFs) has been reported in several European countries, including Italy [7–13]. This non-B subtypes increase occurred in conjunction with the relevant epidemiological changes such as the migratory waves from low-middle income areas [14,15], and the increase in the homosexual transmission route [16–18], with men who have sex with men (MSM) accounting for at least 43% of these new HIV-1 cases [1,14,19–21].

Thus, despite the effort to control the transmission of HIV-1 through antiretrovirals and prevention strategies, HIV-1 infection remains a major public health issue in Europe, with evidence of relevant epidemic transmission in several European countries [2–4,7,22].

To date, phylogenetic analysis represents one of the most important tools to better describe and monitor local HIV epidemics, by correlating the genetic relationship of the viruses with information on demographics, transmission mode, new infections and drug resistance.

We present two recent HIV-1 transmission clusters among newly diagnosed Italian men, engaging in high-risk behaviours, including MSMs and a small proportion of men who have sex with men and women (MSMW). All individuals were infected by HIV-1 non-B subtypes carrying NNRTI-related mutations and were naïve to antiretroviral drugs. These events can explain the role of high-risk behaviours for HIV-1 transmission in the ongoing change of the HIV-1 epidemic in Italy, and the role of molecular epidemiology in prevention efforts.

## Methods

### Study Population

All patients included in the analyses were individuals with HIV-1 infection confirmed in different counselling and testing (CT) centres in Central Italy, between May 2011 and September 2014, as a part of SENDIH study, a regional prospective, multi-centre observational study collecting socio-demographic, behavioural, clinical and virologic characteristics on new HIV diagnoses [23].

Patients were defined as recently infected by: i) clinical signs of primary HIV infection (HIV-1 RNA levels >10,000 copies/mL and negative or indeterminate HIV-1 antibody test); ii) a documented negative HIV-1 test within six months before the HIV-1 diagnosis; iii) laboratory evidence (avidity index ≤ 0.8) of a seroconversion during the six months preceding the HIV-1 diagnosis [24,25].

All clinical and virological information used in this study was collected within 8 weeks after the initial HIV-1 diagnosis (min-max weeks after HIV-1 diagnosis: 0–8).

### Ethics Statements

The SENDIH (Studio Epidemiologico Nuove Diagnosi Infezione HIV-1) study was approved by the ethics committee of the L. Spallanzani National Institute for Infectious Diseases in 2003 (Ethics Approval N° 51, in date 2003, December 18). All HIV-1 newly diagnosed individuals filled out a behavioural questionnaire, and provided written informed consent for getting permission to use the collected epidemiological and virological information, including HIV sequences. All the information collected during the study is recorded in an electronic database after coding all personal identifiers to guarantee patients’ anonymity and to prevent patients’ identification. A copy of the Ethical Approval was also included as (S1 Appendix).

### HIV-1 Genotyping

For all patients HIV-1 *pol* (containing the full-length protease [PR] and the first 335 reverse transcriptase [RT] codons) and V3 sequences were available at the time of diagnosis (range 0–2 months from diagnosis). HIV-1 *pol* and V3 genotype analyses were performed on plasma samples, as previously described [26,27]. All samples were processed as soon as they arrived in clinical laboratories.

### HIV-1 Subtyping Assignment

For each patient, HIV-1 subtype was determined. *Pol* sequences were aligned and compared with reference sequences for the major HIV-1 subtypes, available at: http://hiv-web.lanl.gov/content/hiv-db/SUBTYPE_REF/align.html using CLUSTAL X. The sequences were then manually edited with the Bioedit program, and gaps were removed from the final alignment. Subtype or CRF assignments were achieved by constructing phylogenetic trees using the Neighbor-Joining (NJ) method [28]. Distances were calculated using MEGA 6 based on the Kimura-2 parameter (K2P) model [29]. The reliability of the branching orders was assessed by bootstrap analysis of 1000 replicates. Subtype classification was also confirmed by the REGA HIV-1 subtyping tool, COMET subtype tool [30], and DataMonkey subtype tool. To improve the accuracy of recombinant and unique forms, RDP3 software and Splits Tree software were used.

### Identification of Transmission Clusters

Once the subtypes were assigned, transmission clusters were first deduced by the NJ method evaluated by an initial dataset containing all *pol* sequences obtained for routine clinical practice between January 2000 and December 2014 from both naïve and drug-experienced individuals infected by non-B subtypes. The dataset also contained the specific subtype reference sequences. All reference sequences were downloaded from http://www.hiv.lanl.gov/content/sequence/NEWALIGN/align.html. To avoid any possibility of cross-contamination, identical sequences amplified in the same run were excluded. In addition, once the phylogenetic analysis highlighted the presence of transmission clusters, all samples were repeated one more time in 2 independent laboratories. To avoid the influence of convergence evolution at antiretroviral drug resistance mutations, sequences were stripped. Only clusters with a bootstrap value higher than 90% and an average genetic distance <0.015 were selected [31].

The robustness of the transmission clusters was further tested using the Maximum Likelihood (ML) method and a Bayesian analysis by including as control a subgroup of *pol* sequences, randomly selected from those used for the NJ tree.

The ML tree was inferred with the General Time-Reversible nucleotide substitution model (GTR) with gamma-distribution among site rate heterogeneity, a proportion of invariable sites (GTR+I+Г_5_) [32], and 1,000 bootstrap replicates (using the PhyML program, available at http://www.atgc-montpellier.fr/phyml/). Transmission clusters were identified by a bootstrap support >90%. The tree was rooted using a midpoint rooting by FigTree software version 1.4.2.

The Bayesian phylogenetic tree was reconstructed with MrBayes [33], using a GTR+I+Г_5_. The Monte Carlo Markov Chain (MCMC) search was run for 5x10^6^ generations with the trees sampled every 100th generation (with a burn-in of 50%) [34]. Statistical support was obtained by calculating the posterior probability of each monophyletic clade, and a posterior consensus tree was generated after 50% burn-in. Clades were considered epidemiological clusters only if a posterior probability of 1 was inferred.

### Estimation of Evolutionary Rates and Dates

The dated trees, evolutionary rates and population growth were co-estimated by using a Bayesian MCMC approach (BEAST software package 1.8.1), implementing GTR+I+ Г model [35]. For this analysis, the same subgroup of randomly selected *pol* sequences used for the ML and Bayesian analysis, within the HIV-1 *pol* sequences involving in the clusters, was used. These sequences were assembled in two different datasets according to the specific subtype.

As coalescent priors, different parametric demographic models (constant population size, exponential and logistic growth) and a non-parametric Bayesian skyline plot (BSP) were compared under strict and relaxed clock conditions (log-normal). The best combination of models was selected after testing several alternative models for each prior category, by calculating the Bayes factor (BF) with TRACER version 1.6 [36].

MCMC simulations were run for 50 x 10^6^ steps, sub-sampling parameters every 1,000 steps, with a 10% burn-in. Convergence of parameters was assessed by calculating the Effective Sample Size (ESS) using TRACER version 1.6, after excluding an initial 10% for each run. All parameter estimates for each run showed ESS values >250. The trees were summarized in a target tree by the Tree Annotator program included in the BEAST package by choosing the tree with the maximum product of posterior probabilities (maximum clade credibility) after a 50% burn-in. The tree was rooted using a midpoint rooting by Fig-Tree software version 1.4.2.

### Resistance Analysis and Tropism Prediction

HIV-1 strains were defined as resistant if carrying at least one drug resistance mutation among the mutations listed by Bennett et al [37]. and the primary mutations reported in the IAS-USA list (IAS 2014) and/or the HIV Drug Resistance Stanford Database (http://hivdb.stanford.edu/). Polymorphisms at positions already associated with drug resistance were also investigated.

HIV-1 co-receptor usage was inferred from the V3 nucleotide sequence by using the Geno2Pheno algorithm available at the following website: http://coreceptor.bioinf.mpi-inf.mpg.de/.

The analysis was performed setting Geno2Pheno at false positive rate (FPR) of 10%. Thus, sequences with FPR <10% were considered X4/DM tropic [38].

### Statistical Analyses

Differences regarding the epidemiological characteristics between the two clusters were evaluated as follows: i) for the categorical variables, by Fisher’s exact test ii) for the continuous variables, by the Kruskal-Wallis test. In all the analyses performed, *P values* <0.05 were considered as statistically significant. The statistical program used was SPSS (version 19) for Windows (SPSS Inc., Chicago, Illinois).

## Results

### Clusters Identification

Among 1,546 new cases of HIV-1 infections diagnosed between May 2011 and September 2014 in different counselling and testing (CT) centres in Central Italy, 534 (34.5%) belonged to non-B subtypes. Demographics and viro-immunological characteristics of these 534 patients were reported in (S1 Table).

Among the 534 HIV-1 non-B infected patients, 35 drew our attention, because by a preliminary phylogenetic analysis by the NJ method, performed on 2,158 *pol* non-B subtype sequences obtained for routine clinical practice between January 2000 and December 2014, their HIV-1 *pol* sequences (27 belonged to C subtype and 8 to CRF17_BF subtype) formed two distinct clusters (bootstrap >90%).

**Fig 1:**
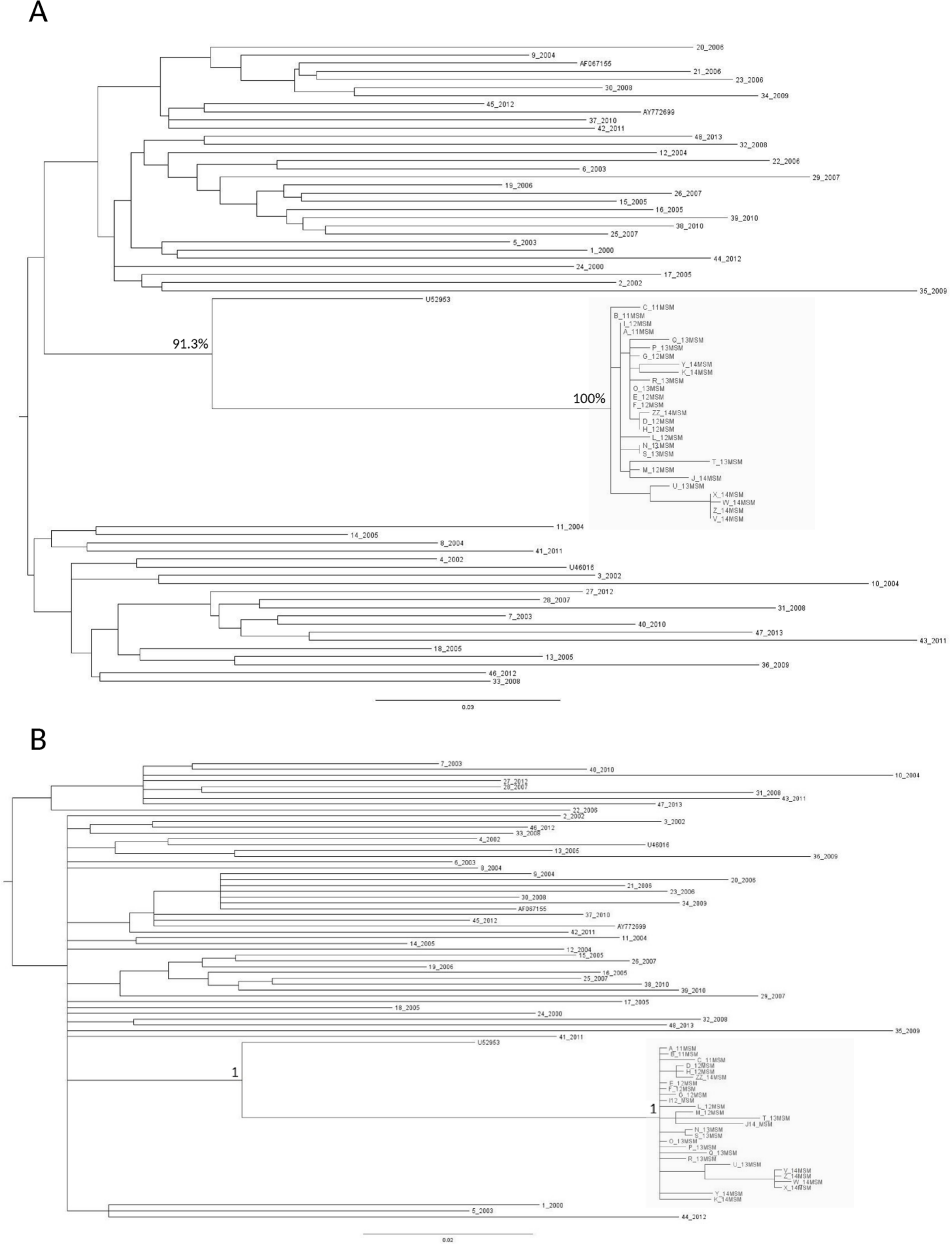
Maximum likelihood (ML) tree of the 75 C sequences plus the 4 C references (A). The ML tree was inferred by using PhyML program. Transmission clusters were identified by a bootstrap support >90% (clusters defined by the grey box). The tree was rooted using a midpoint rooting. Bayesian phylogenetic tree of the 75 C sequences plus the 4 C references (B). The Bayesian phylogenetic tree was inferred by using MrBayes. Clades with a posterior probability of one were considered epidemiological clusters (defined by the grey box).

**Fig 2:**
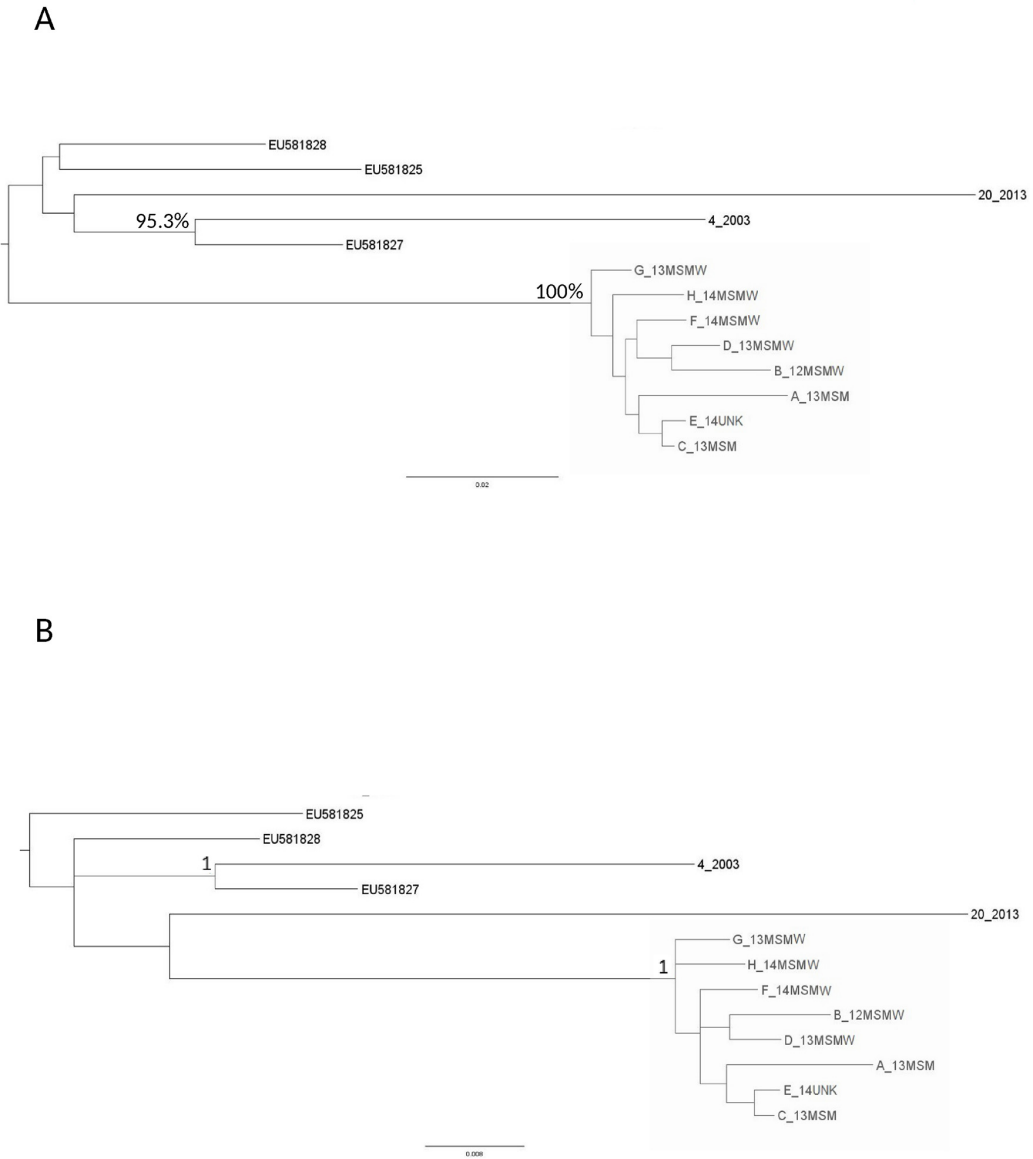
Maximum likelihood (ML) tree of the 10 CRF17_BF sequences plus the 3 CRF17_BF references (A). The ML tree was inferred by using PhyML program. Transmission clusters were identified by a bootstrap support >90% (clusters defined by the grey box). The tree was rooted using a midpoint rooting. Bayesian phylogenetic tree of the 10 CRF17_BF sequences plus the 3 CRF17_BF references (B). The Bayesian phylogenetic tree was inferred by using MrBayes. Clades with a posterior probability of one were considered epidemiological clusters (defined by the grey box).

The statistical robustness of both clusters was confirmed by the ML and the Bayesian phylogenetic trees. These trees were performed using 75 C and 10 and CRF17_BF *pol* sequences, including the 35 sequences identified in the preliminary phylogenetic analysis and 50 *pol* sequences (48 belonged to the C subtype and 2 belonged to CRF17_BF) randomly selected from the 2,158 obtained for routine clinical practice (Figs 1 and 2).

Both clusters were characterized by a bootstrap value of 100% (Figs 1A and 2A), and a posterior probability of one (Figs 1B and 2B).

An additional confirmation of the high homology among sequences involved in the two clusters arose from the extremely low mean genetic distance (± standard error [SE]) observed for the *pol* gene in both C (0.00608±0.00109) and CRF17_BF clusters (0.00989±0.00198).

The results obtained by the phylogenetic analyses on the *pol* gene were also confirmed by using the V3 loop, despite the short length of the region (only 105 nucleotides) and its higher variability compared to *pol* gene (V3 mean genetic distance: 0.00849±0.00452 for C cluster, and 0.12999±0.04550 for CRF17_BF cluster) (data not shown).

### Clusters Dating

In order to obtain the time of origin of HIV-1 C and CFR17_BF clusters, the evolutionary rate based on known sampling date of our sequences was estimated by a Bayesian MCMC approach. The trees included the 35 sequences involved in the clusters and additional 50 *pol* sequences, of which 48 belonged to the C subtype and 2 to the CRF17_BF, selected from the same dataset mentioned before.

**Fig 3:**
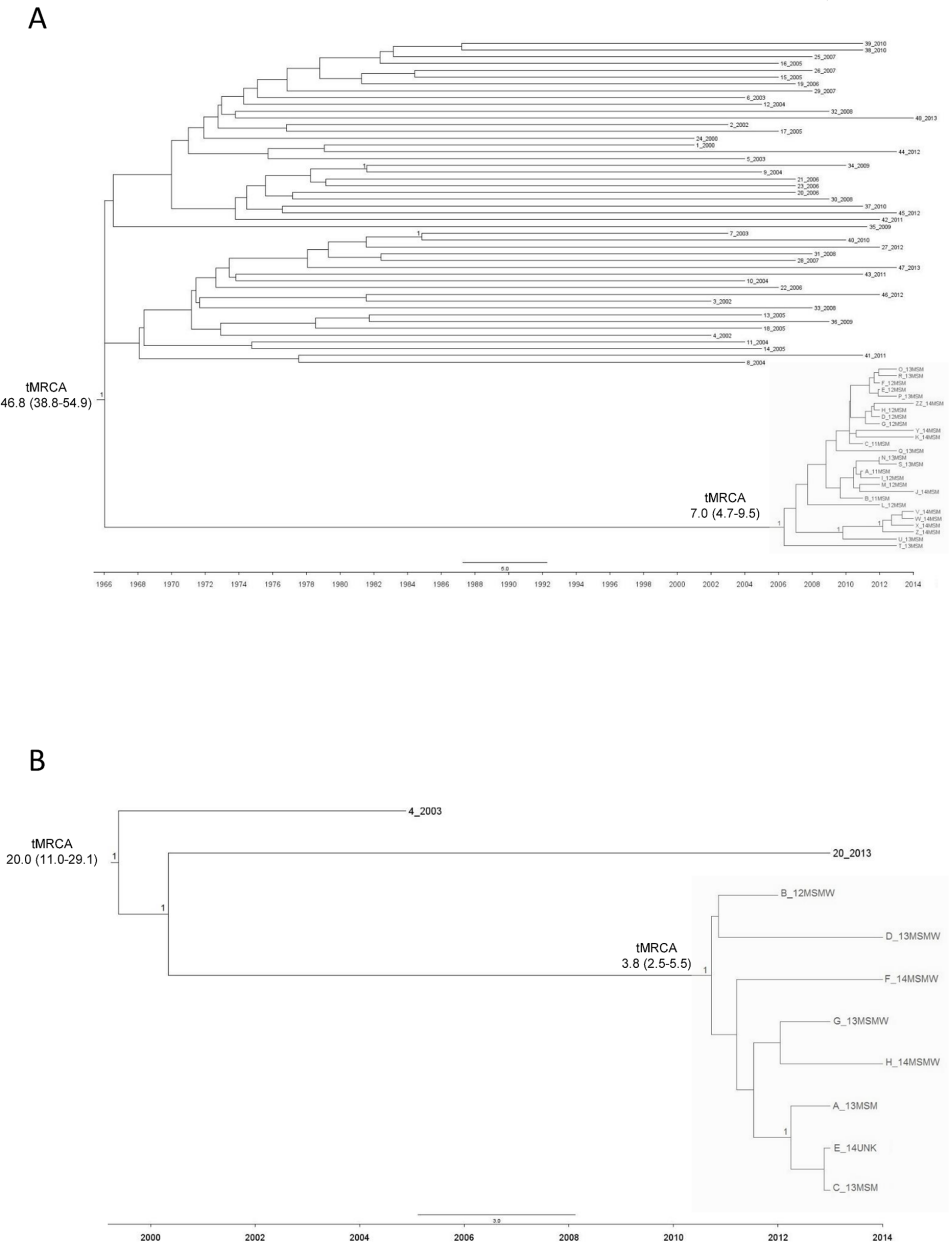
Bayesian time-scaled phylogenetic tree of the HIV-1 subtype C sequences (A) and CRF17_BF (B). The trees were generated under a relaxed molecular clock model using BEAST. The X-axis of the tree represents time (in years). A posterior probability of one is positioned along the branches. The clusters involving the 27 HIV-1 C sequences and the 8 HIV-1 CRF17_BF sequences were in the grey boxes. tMRCA: time of the Most Recent Common Ancestor.

Fig 3 shows dated trees for both C and CRF17_BF sequences (Fig 3). The trees are scaled by calendar year, from which the period of cluster transmissions can be inferred.

For the Bayesian phylogenetic tree, a relaxed clock with an uncorrelated log-normal rate distribution, assuming the GTR+I+Г_5_ model of nucleotide substitution, was performed for both clusters. As coalescent priors, parametric demographic models (logistic growth for C subtype and constant size for CRF17_BF) were used. The rate of evolution at *pol* gene previously estimated (2.5x10^-3–^1.8x10^-3^ substitution/site/year) [21,39] was incorporated as a prior probability distribution (log-normal prior). The best combination of models was selected after testing several alternative models for each prior category, by calculating the Bayes factor (BF) with TRACER version 1.6 [36].

First, a mean evolutionary rate was estimated for both C (mean [95% HPD]: 1.45x10^-3^ [1.19x10^-3^–1.72x10^-3^]), and CRF17_BF sequences (mean [95% HPD]: 2.70x10^-3^ [1.45x10^-3^–4.13x10^-3^]). Because of these evolutionary rates, the time of the Most Recent Common Ancestor (tMRCA) was about 46.8 years before 2014 (95% HPD: 38.8–54.9) for the 75 HIV-1 subtype C sequences analysed (Fig 3A), and 20.0 years before 2014 (95% HPD: 11.0–29.1) for all the 10 HIV-1 CRF17_BF sequences (Fig 3B).

Focusing the attention on the C cluster, the mean time of the MRCA of the cluster was 7.0 years before 2014 (95% HPD: 4.7–9.5); thus, the origin of this cluster can be traced around 2007 (95% HDP: 2004–2009) (Fig 3A). A most recent tMRCA was found for the CRF17_BF cluster. In particular, patients involved in this cluster had a mean tMRCA of 3.8 years (95% HPD: 2.5–5.5), thus letting us hypothesize that this cluster was originated in the last months of 2010 (95% HDP: 2008–2011) (Fig 3B).

### Epidemiological Characteristics of HIV-1 C and CRF17_BF Clusters

Epidemiological characteristics of the 35 newly diagnosed HIV-1 individuals involved in the two clusters are reported in Table 1. All of them were men and Italians, with the exception of one patient, who belonged to CRF17_BF subtype, coming from Argentina. Regarding risk factors, while the C cluster was entirely composed by Italian MSM, 5/8 (62.5%) men involved in the CRF17_BF cluster reported sexual contacts with men and women. Interestingly, all these 5 men reporting bisexual behaviours had female steady partners who were promptly screened for HIV. None of these women was identified as HIV positive.

**Table 1:** Details of patients involving in the HIV-1 C and CRF17_BF epidemiological clusters circulating in Central Italy. Abbreviations: FPR, False Positive Rate; MSM, Men who have Sex with Men; MSMW, Men who have Sex with Men and Women; UNK, Unknown; NA, Not Available; NNRTI, Nucleoside Reverse Transcriptase Inhibitors; NRTI, Nucleos(t)ide Reverse Transcriptase Inhibitors; PI, Protease Inhibitor

Cluster	Sequence	Sampling Date	Risk group	NRTI resistance mutation	NNRTI resistance mutation	PI resistance mutation	Atypical Mutation	V3 FPR	Sex	Age	Viral Load (log_10_copies/mL)	CD4 (cells/mm3)	Nation of Origin	Recent Infection
C	A_11MSM	2011	MSM	-	-	-	K103Q	48.6	Male	29	6.90	721	Italy	Yes
C	B_11MSM	2011	MSM	-	-	-	K103Q	17.0	Male	31	5.07	901	Italy	Yes
C	C_11MSM	2011	MSM	-	-	-	K103Q	14.3	Male	40	4.80	662	Italy	-
C	D_12MSM	2012	MSM	-	-	-	K103Q	14.3	Male	37	5.16	469	Italy	-
C	E_12MSM	2012	MSM	-	-	-	K103Q	14.3	Male	37	4.85	547	Italy	-
C	F_12MSM	2012	MSM	-	-	-	K103Q	14.3	Male	31	6.39	626	Italy	Yes
C	G_12MSM	2012	MSM	-	-	-	K103Q	15.4	Male	29	5.17	365	Italy	Yes
C	H_12MSM	2012	MSM	-	-	-	K103Q	14.3	Male	27	5.33	638	Italy	-
C	I_12MSM	2012	MSM	-	-	-	K103Q	14.3	Male	37	5.28	352	Italy	-
C	L_12MSM	2012	MSM	-	-	-	K103Q	14.3	Male	26	5.79	289	Italy	-
C	M_12MSM	2012	MSM	-	-	-	K103Q	14.3	Male	34	5.45	490	Italy	-
C	N_13MSM	2013	MSM	-	-	-	K103Q	14.3	Male	51	5.90	643	Italy	-
C	O_13MSM	2013	MSM	-	-	-	K103Q	14.3	Male	27	5.11	291	Italy	-
C	P_13MSM	2013	MSM	-	-	-	K103Q	44.5	Male	30	4.60	588	Italy	-
C	Q_13MSM	2013	MSM	-	-	-	K103Q	14.3	Male	44	4.56	503	Italy	Yes
C	R_13MSM	2013	MSM	-	-	-	K103Q	15.4	Male	37	5.72	227	Italy	-
C	S_13MSM	2013	MSM	-	-	-	K103Q	14.3	Male	48	5.02	488	Italy	-
C	T_13MSM	2013	MSM	-	-	-	K103Q	14.3	Male	32	5.03	389	Italy	-
C	U_13MSM	2013	MSM	-	-	-	K103Q	16.4	Male	33	5.12	316	Italy	-
C	V_14MSM	2014	MSM	-	-	-	K103Q	16.4	Male	49	5.76	443	Italy	-
C	Z_14MSM	2014	MSM	-	-	-	K103Q	15.4	Male	40	4.15	387	Italy	Yes
C	Y_14MSM	2014	MSM	-	-	-	K103Q	15.4	Male	25	3.69	959	Italy	Yes
C	W_14MSM	2014	MSM	-	-	-	K103Q	14.3	Male	34	6.38	596	Italy	Yes
C	X_14MSM	2014	MSM	-	-	-	K103Q	15.4	Male	39	5.08	760	Italy	Yes
C	K_14MSM	2014	MSM	-	-	-	K103Q	13.2	Male	33	4.22	870	Italy	Yes
C	J_14MSM	2014	MSM	-	-	-	K103Q	15.4	Male	26	6.43	355	Italy	Yes
C	ZZ_14MSM	2014	MSM	-	-	-	K103Q	14.3	Male	55	5.32	368	Italy	-
CRF17_BF	A_13MSM	2013	MSM	-	K101E/E138K	-	-	25.3	Male	50	2.59	584	Italy	Yes
CRF17_BF	B_12MSMW	2012	MSMW	-	K101E/E138K	-	-	96.2	Male	49	5.14	347	Italy	-
CRF17_BF	C_13MSM	2013	MSM	-	K101E/E138K	-	-	96.2	Male	28	5.49	199	Argentina	-
CRF17_BF	D_13MSMW	2013	MSMW	-	K101E/E138K	-	-	96.2	Male	65	6.03	591	Italy	Yes
CRF17_BF	E_14UNK	2014	UNK	-	K101E/E138K	-	-	25.3	Male	60	4.22	689	Italy	-
CRF17_BF	F_14MSMW	2014	MSMW	-	K101E/E138K	-	-	25.3	Male	52	4.71	195	Italy	-
CRF17_BF	G_13MSMW	2013	MSMW	-	K101E/E138K	-	-	96.2	Male	44	4.55	414	Italy	-
CRF17_BF	H_14MSMW	2014	MSMW	-	K101E/E138K	-	-	96.2	Male	58	4.80	459	Italy	-

By comparing age, we found that patients in the CRF17_BF cluster were more likely to be older than those involved in the C cluster, and than patients who belonged to the other 499 non-B new diagnoses not involved in clusters. In particular, the median age was 51 (IQR: 48–59) years in the CRF17_BF cluster, 34 (IQR: 30–39) years in the C cluster, and 36 (IQR: 29–43) years in the other HIV-1 non-B infections (p = 0.02).

By comparing viro-immunological parameters, clusters were characterized by a viral load similar to that observed for the 499 new HIV-1 non-B diagnoses (median [IQR)] 5.16 [4.93–5.74] log_10_ copies/mL in C cluster versus 4.76 [4.47–5.32] log_10_ copies/mL in CRF17_BF cluster, versus 5.02 [4.40–5.60] in the other new cases, p = 0.15). By contrast, CD4 cell counts were significantly higher in patients involved in the clusters than in the other 499 new diagnoses (490 [366–640] cells/mm^3^ in the C cluster versus 436 [310–585] cells/mm^3^ for the CRF17_BF cluster, versus 297 [129–469] in the other new diagnoses, p = 0.036), highlighting that patients involved in clusters arrived earlier than the others at the diagnosis.

The early HIV-1 diagnosis for patients involved in the clusters can be also explained by the frequency and motivations for HIV-1 testing reported in the anamnesis. Interestingly, among the 23 patients for those this information is available, 21 (91.3%) reported at least one HIV negative test within their lifetime, and for 13 of them (7 for cluster C and 6 for cluster CRF17_BF) this test was performed during the last year before HIV-1 diagnosis. The most commonly reported motivation for HIV testing was a self-perception of clinical symptoms related to HIV infection (52.2%, 8 individuals for C cluster and 4 for CRF17_BF cluster), followed by the behaviour to perform routine HIV-testing (every six months) (17.4%, 4 individuals, all belonged to C cluster). It is also important to note that 13 patients involved in the clusters (37.1%) were classified as recently infected (11/27, 40.7% within HIV-1 C cluster, and 2/8, 25.0% within CRF17_BF cluster).

### Drug Resistance and Tropism Prediction

Information about HIV-1 drug resistance and tropism prediction for each one of the 35 patients analysed has been reported in Table 1.

All HIV-1 strains in the C cluster carried the rare and atypical RT mutation K103Q (prevalence in non-B subtypes: 1.6% and 1.1% for drug-naïve and drug-experienced patients, respectively, personal data), indicating the sharing of a virus characterized by a mutation at a position critical for Nevirapine and Efavirenz efficacy. All viruses were R5-tropic, and were characterized by the V3 mutations H13R and E25D, known to be significantly associated with CXCR4 and CCR5 usage, respectively [40]. Focusing the attention on FPR, the majority (25/27) of patients were infected by an HIV-1 virus with an FPR around 15%, while two patients carried a virus with an FPR of 48.6% and 44.5% (A_11MSM and P_13MSM). This high FPR value can be explained by the presence of a single amino-acid change at position 10 of V3 region (K10E).

Analysing the HIV-1 CRF17_BF cluster, all strains carried the NNRTI resistance mutations K101E and E138K in the RT, thus showing the transmission of a resistant viral strain.

Regarding the V3 tropism prediction, patients in this cluster were infected by R5 viruses, such as patients in the C cluster. However, by contrast with the HIV-1 C cluster (where V3 sequences were characterized by a similar FPR) in the CRF17_BF cluster the FPR values ranged from 25.3% to 96.2%. In particular, three patients carried strains characterized by an FPR of 25.3%, and by the X4 markers E25Q and Q32K. By contrast, five patients carried HIV-1 strains characterized by a very high FPR (96.2%), explained by the presence of a double amino-acid change at 22 and 25 positions of the V3 region (T22A and E25D), known to be significantly associated with a CCR5 coreceptor usage [40], and the absence of the positive charge at position 32.

## Discussion

By combining traditional epidemiological data and more recently developed bio-molecular analyses, we were able to define, in a restricted geographical area of Central Italy, two HIV-1 transmission clusters characterized by common features. Both clusters involved newly diagnosed individuals infected by non-B HIV-1 strains (27 C and 8 CRF17_BF) with evidence of atypical and transmitted drug-resistance related to NNRTI-drugs; all individuals were men with high risk behaviours (MSM and MSMW). All of them were Italian, with the only exception of one patient from Argentina. A considerable proportion of them were in recent infection, particularly in the C cluster, and most of them were early diagnosed as indicated by the high CD4 cell count. In addition most of them reported prior HIV negative testing, and were mainly motivated by the need to check routinely their health-status. Overall these data may suggest that individuals involved in these clusters have a high self-perception of risk for acquiring HIV, and for this reason arrived early at the diagnosis.

Despite the common features, there are a few differences characterizing the two clusters. In particular, only patients involved in the CRF17_BF cluster reported being MSMW. These patients were also older than the C cluster individuals, confirming that homosexual orientation is frequently masked by bisexual behaviours in older individuals [21,41].

Overall, these findings confirm that, despite the prevention strategies implemented for the limitation of HIV infection, the rapid spread of HIV can still occur, especially among men engaging in high-risk behaviours, and frequently involves new and rare recombinant forms of HIV-1. Even if this concept has also been reported in several recent studies that highlighted the role of homosexual and non-B subtypes in the circulation of HIV-1 [20–22,42], this is the first time that clusters of noteworthy relevance have been described in Italy. Moreover, these findings are suggestive of multiple introduction of non-B variants with a high rate of transmission, particularly in patients with high-risk behaviours including multiple sexual partners, a low rate of condom usage, more opportunities for sexual relationships, and a low HIV detection rate. By considering the bisexual behaviours reported by several individuals who belonged to CRF17_BF cluster, HIV transmission to heterosexual women should not be ruled out.

The dated phylogenies reconstruction highlights the recent origin of these two clusters, traced after 2007 for both clusters.

In particular, the origin of the C cluster was estimated around 2007. It is known that the C subtype is one of the first non-B subtypes described in Italy, and is responsible for a significant part of the HIV-1 non-B epidemic in this country [10,43]. The circulation of this HIV-1 subtype in Europe occurred at the beginning of the seventies, thirty years later than its origin reported in Africa [39,43]. This datum is also confirmed by our dated phylogenetic reconstruction, with a tMRCA of about 46.8 years before 2014. Subtype C is in general prevalently associated with immigrants from South America and sub-Saharan Africa, especially from the Southern region. The cluster here reported shows instead an ongoing circulation of the C subtype among Italian individuals, and suggests that the spread of the C subtype in Italy is to date also dependent on Italian subjects, as indicated by the lack of a foreign individual in this cluster.

The second cluster described in this paper involved individuals infected by the uncommon CRF17_BF recombinant form. This CRF belonged to the BF inter-subtype recombinants, that were almost exclusively found in South America, and in European countries with a social and cultural exchange with Latin America like Spain and Italy [44]. This CRF was first identified in 2001 in Argentina, and up to the present has been found in less than 2% of the total HIV-1 BF recombinant forms in this country [45]. Interestingly, the presence in our cluster of a subject from Argentina let us suppose that the common source of infection may have originated just from that country. Our dated phylogenetic reconstruction confirms the recent appearance of this recombinant form in Italy, tracing the origin of this cluster around 2010, and the origin of this CRF in the nineties. In this regard, a potential limitation of this finding is the limited number of CRF17_BF sequences available for the analyses. Further studies with more sequences are needed to confirm this result.

The phylogenetic clustering highlighted the existence of viral lineages characterised by NNRTI related mutations involving only drug naïve individuals.

Indeed, all patients involved in the HIV-1 C cluster carried HIV-1 strains with the K103Q, an atypical and rarely found mutation present in a position critical for Nevirapine and Efavirenz efficacy. This mutation is codified by the CAA (Q) codon that decreases the genetic barrier to select the NNRTI drug resistance K103H mutation [46]. Thus, even if this mutation is already known by the literature to not confer NNRTIs resistance [46], we can hypothesize that this atypical RT mutation can represent a revertant for K103H. In this regard, further investigations are needed to deeply characterize this mutation and its role in drug resistance. By contrast, the CRF17_BF cluster has been characterized by HIV-1 strains resistant to both first and second NNRTIs generations, due to the presence of the RT mutations K101E and E138K [47]. These mutations are rarely found in drug-naïve HIV infected patients (prevalence: <0.4% for both non-B and B subtypes strains) [48], but were selected in a high proportion of patients receiving NNRTIs [49].

These findings suggest that also non-B subtypes in Italy can have a primary role in the spread of drug resistance, to date mainly associated with the B subtype. Indeed, complex transmission clusters carrying drug resistance strains have to date been observed mainly in the context of B subtype infections [22,50,51].

It should be noted that, although the phylogenetic analysis was performed including sequences from both drug-naïve and drug-experienced patients, the two identified clusters were exclusively composed by sequences from drug-naïve individuals. However, we cannot rule out the involvement of drug-experienced individuals in the clusters for which the *pol* sequences were not available.

The analysis of V3 sequences revealed that all patients in the clusters were infected by an R5 tropic virus. However, V3 loops were characterized by different FPR values also in the setting of the same cluster (from 14.3% to 48.6% in C cluster, and 25.3% and 96.2% in CRF17_BF cluster). This can be probably due to different mutational patterns characterizing the V3 loop of these patients. In particular, in the setting of the C cluster, all the 27 viruses carried key mutations required for both CCR5 and CXCR4 binding, such as the E25D and H13R [40,52]. The analysis of the V3 region of patients involved in the CRF17_BF cluster revealed, instead, the existence of two distinct viral species, both R5 by the Geno2Pheno algorithm, the first one characterized by the CCR5 key mutations T22A and E25D, and the other one by the CXCR4 associated mutations E25Q and Q32K [40,52]. Thus, it is conceivable that these two viral species (prevailing in five and three patients, respectively) may coexist in all patients involved in the cluster. Overall, these findings confirm the elevated complexity and the high degree of variability in the V3 region, crucial for the coreceptor choice in the setting of epidemiological clusters. Our findings also support the use of the genotypic test in newly diagnosed patients, which remains the cornerstone for clinicians to set-up and individualize initial therapy especially in patients infected with resistant HIV-1 strains, such as those analysed in this study. It is conceivable that in these specific patients the drop and long-term maintenance of viral load below 50 copies/mL can be guaranteed only by using a combination of potent drugs, not including NNRTIs, but belonging to protease-inhibitors or new drug classes (like integrase inhibitors or maraviroc, considering the prevalent R5 tropism of viral strains harbouring in these patients) [53].

Finally, we want to highlight the role of molecular tests to support traditional epidemiology, to characterize highly connected HIV-1 transmission clusters, and to better understand dynamics of HIV-1 transmission. These data, mainly those regarding recently acquired infections, could be used by local public health officials to better allocate available resources for successful interventions for prevention.

Our findings, in fact, provide the first evidence of a strong and recent circulation in central Italy of non-B subtypes clusters carrying NNRTI-related amino acidic mutations, among newly diagnosed Italian men engaging in high-risk behaviours. This implies that an improvement of HIV-1 prevention strategies and screening activities, especially in the setting of a population at high risk for HIV is needed, such as the earlier detection of HIV infection, and the earlier beginning of antiretroviral treatment, as recommended in the most recent treatment guidelines [54,55].

### Nucleotide Sequence Accession Number

The 35 *pol* sequences involved in the two HIV-1 transmission clusters have been submitted to GenBank under accession numbers from KT343868 to KT343902.

## Supporting Information

S1 Table Characteristics of the 534 HIV-1 newly diagnosed patients. (DOCX)

S1 Appendix Ethics Approval of the SENDIH study (Studio Epidemiologico Nuove Diagnosi Infezione HIV-1). (PDF)
