# Supplementary material for: Recent Transmission Clustering of HIV-1 C and CRF17_BF Strains Characterized by NNRTI-Related Mutations among Newly Diagnosed Men in Central Italy
Source: PLoS One. 2015 Aug 13;10(8):e0135325. doi: 10.1371/journal.pone.0135325 (PMC4535860; doi:10.1371/journal.pone.0135325)
Supplement: S1 Table — (DOCX) [file pone.0135325.s001.docx]

**S1 Table.** Characteristics of the 534 HIV-1 newly diagnosed patients.

| Characteristics | Overall (N=534) | Patients involved in the 2 clusters (N=35) | Patients not involved in the 2 clusters (N=499) | p-value^a^ |
| --- | --- | --- | --- | --- |
| Male, n (%) | 387 (72.5) | 35 (100) | 352 (70.5) | 0.005 |
| Age (median, IQR) | 36 (30-46) | 37 (30-49) | 36 (29-43) | 0.83 |
| Italian, n (%) | 260 (48.7) | 34 (97.1) | 226 (45.3) | <0.001 |
| Year of diagnosis,  (median, IQR) | 2012 (2011-2013) | 2013 (2012-2014) | 2012 (2011-2013) | 0.01 |
| Risk factor, n (%) |  |  |  |  |
| Heterosexual | 192 (36.0) | 0 (0.0) | 192 (38.5) | <0.001 |
| MSM | 120 (22.4) | 29 (82.9) | 91 (18.2) | <0.001 |
| MSMW | 49 (9.2) | 5 (14.3) | 44 (8.8) | 0.36 |
| Injection Drug Users | 18 (3.4) | 0 (0.0) | 18 (3.6) | 0.25 |
| Other/Unknown | 155 (29.0) | 1 (2.8) | 154 (30.9) | <0.001 |
| Recent Infection^b^, n (%) | 31 (29.2) | 13 (37.1) | 18 (25.3) | 0.33 |
| At diagnosis: |  |  |  |  |
| Viral load, log_10_ copies/mL (median, IQR) | 5.0 (4.4-5.6) | 5.1 (4.8-5.6) | 5.0 (4.4-5.6) | 0.21 |
| CD4 cell count, cells/mm^3^ (median, IQR) | 321 (158-507) | 488 (360-632) | 297 (129-469) | <0.001 |
| Subtype, n (%): |  |  |  |  |
| F1 | 123 (23) | - | 123 (24.7) | - |
| G | 34 (6.4) | - | 34 (6.8) | - |
| C | 88 (16.5) | 27 (77.1) | 61 (12.2) | <0.001 |
| A1 | 36 (6.7) | - | 36 (7.2) | - |
| K | 7 (1.3) | - | 7 (1.4) | - |
| D | 5 (0.9) | - | 5 (1.0) | - |
| CRF02_AG | 129 (24.1) | - | 129 (25.9) | - |
| CRF31_BC | 14 (2.6) | - | 14 (2.8) | - |
| CRF12_BF | 35 (6.6) | - | 35 (7.0) | - |
| CRF01_AE | 18 (3.4) | - | 18 (3.6) | - |
| CRF17_BF | 10 (1.9) | 8 (22.9) | 2 (0.4) | <0.001 |
| CRF06_cpx | 10 (1.9) | - | 10 (2.0) | - |
| CRF14_BG | 4 (0.7) | - | 4 (0.8) | - |
| CRF28_BF | 3 (0.6) | - | 3 (0.6) | - |
| CRF03_AB | 3 (0.6) | - | 3 (0.6) | - |
| Other | 15 (2.8) | - | 15 (3.0) | - |
| X4 tropism (FPR<10%)^c^ | 26 (7.5) | 0 (0.0) | 26 (8.4) | 0.14 |
| At least one drug resistance mutation, n (%) | 43 (8.6) | 8 (22.9) | 35 (7.0) | 0.05 |

Abbreviations: MSM, Men who have Sex with Men; MSMW, Men who have Sex with Men and Women; FPR, False Positive Rate. ^a^Statistically significant differences between the 35 patients involved in the clusters C and CRF17_BF and the 499 not involved in the clusters were assessed by Chi square test for categorical variables and by Wilcoxon test for continuous variables. ^b^The infection status (recent or chronic) was known for 106 patients (35 involved in the clusters, and 71 not involved in the clusters). ^c^Information about HIV-1 tropism was available for a total of 345 patients (35 involved in the clusters, and 310 not involved in the clusters).
