# Supplementary material for: Recent Transmission Clustering of HIV-1 C and CRF17_BF Strains Characterized by NNRTI-Related Mutations among Newly Diagnosed Men in Central Italy
Source: PLoS One. 2015 Aug 13;10(8):e0135325. doi: 10.1371/journal.pone.0135325 (PMC4535860; doi:10.1371/journal.pone.0135325)
Supplement: S1 Appendix — (PDF) [file pone.0135325.s002.pdf]

ISTITUTO NAZIONALE PER LE MALATTIE [illegible]
"LAZZARO SPALLANZANI" - [illegible]
Via Portuense n. 292 - 00149 ROMA
COMITATO ETICO
COPIA CONFORME ALL'ORIGINALE
COMPOSTA DA N. ...3....... FOGLI
IL SEGRETARIO

Istituto Nazionale per le Malattie Infettive
LAZZARO SPALLANZANI
Istituto di Ricovero e Cura a Carattere Scientifico
Comitato Etico

OGGETTO: **Approvazione nuova versione Protocollo di studio**
TITOLO: "Studio trasversale sulle caratteristiche epidemiologiche e comportamentali delle persone con nuova diagnosi di infezione da HIV"
PARERE N.51 del Registro delle Sperimentazioni

IL COMITATO ETICO
dell'Istituto Nazionale per le Malattie Infettive Lazzaro Spallanzani I.R.C.C.S.
(Istituito con Deliberazione n. 65 del 27/06/96, modificato nella sua composizione da ultimo con la Deliberazione n. 229 del 20/03/2002, il cui funzionamento è disciplinato dal Regolamento adottato con Deliberazione n. 119 del 26/04/1997, da ultimo modificato con Deliberazione n. 390 del 03/08/2000, in possesso dei requisiti di cui al Decreto del Ministro della Sanità del 15 luglio 1997 e del 18 marzo 1998)

RIUNITO IL 18/12/2003

VISTA

La richiesta presentata dal Dott. Enrico Girardi in data 28/11/2003

ESAMINATA

La documentazione fornita a corredo della richiesta stessa, tra cui:
- [x] Protocollo versione n. 2.1 del 26.11.2003
- [x] Sinossi del protocollo
- [x] Modulo di Consenso Informato scritto versione n. 2.1 del 26.11.2003
- [x] Questionario versione n. 2.1 del 26.11.2003

SENTITI

I relatori designati per l'approfondimento dello studio,

VALUTATI

I seguenti aspetti, ove applicabili, ai fini dell'approvazione dello studio:
- Conformità ai principi etici che traggono la loro origine dalle fonti di cui all'art. 2 del Regolamento del Comitato Etico
- Conformità ai principi di Buona Pratica Clinica (GCP) e alle disposizioni normative applicabili
- Conformità ai principi contenuti nel Regolamento delle Sperimentazioni e delle altre ricerche biomediche dell'Istituto Lazzaro Spallanzani
- Rispetto delle regole di riservatezza e confidenzialità previste dalle disposizioni normative applicabili
- Salvaguardia dei diritti, sicurezza e benessere dei soggetti partecipanti allo studio
- Validità scientifica e giustificazione etica dello studio
- Correttezza del disegno sperimentale
- Completezza e chiarezza del modulo di consenso informato e di ogni altra informazione scritta per i soggetti dello studio
- Adeguatezza del rapporto rischi prevedibili/benefici attesi

- Correttezza delle procedure di reclutamento dei soggetti
- Giustificazione delle indagini e/o terapie proposte
- Idonea qualificazione dello sperimentatore e di tutte le persone coinvolte nello studio
- Adeguatezza degli spazi e delle risorse di personale, strutturali e tecnologiche disponibili
- Copertura di tutte le spese aggiuntive che l'Istituto dovrà affrontare per effetto della sperimentazione
- Idonea copertura assicurativa dei soggetti dello studio e degli sperimentatori

APPROVA

La nuova versione del protocollo relativo allo studio in oggetto, da attuarsi presso l'Istituto, sotto la responsabilità scientifica del Dott. Enrico Girardi, Dirigente medico in servizio presso il Dipartimento di Epidemiologia

Si invita, altresì, il Responsabile dello studio a:
- Comunicare per iscritto l'inizio, l'eventuale interruzione e la conclusione dello studio
- Trasmettere una relazione sullo stato di avanzamento dello studio, con periodicità semestrale, e una relazione finale
- Trasmettere eventuali modifiche al Protocollo e/o al modulo di Consenso Informato scritto

Il Presidente del Comitato Etico
(Prof. Ferdinando Dianzani)

Via Portuense, 292 - 00149 Roma Tel. 06.55170705 Fax 06.5594224 E-mail: comitatoetico@inmi.it

Elenco dei membri del Comitato Etico, relative qualifiche e presenze nella seduta del 18/12/2003:

P	A	PRESIDENTE	Prof. Ferdinando DIANZANI	Professore Ordinario di Virologia, Preside Facoltà di Medicina Libera Università Campus Bio-Medico
P	A	VICE PRESIDENTE	Dott.ssa Carlotta MELOCCHI	Coordinatrice Consulta Romana per l'HIV
P	A	COMPONENTE	Prof. Luciano ANGELUCCI	Professore Ordinario di Farmacologia Medica Università degli Studi di Roma La Sapienza
P	A	COMPONENTE	Prof. Ferdinando ANTONIOTTI	Professore Emerito di Medicina Legale fuori ruolo Università degli Studi di Roma La Sapienza
P	A	COMPONENTE	Dott. Pier Luigi BARTOLETTI	Esperto di medicina generale territoriale
P	A	COMPONENTE	Dott.ssa Caterina DI VIGGIANO	Rappresentante Coordinamento Diritti del Cittadino
P	A	COMPONENTE	Dott. Giuseppe IPPOLITO	Direttore Scientifico I.N.M.I. Lazzaro Spallanzani I.R.C.C.S.
P	A	COMPONENTE	Avv. Giuseppe LA GRECA	Presidente aggiunto onorario della Corte Suprema di Cassazione
P	A	COMPONENTE	Dott.ssa Patrizia RELLECATI	Responsabile Servizio Farmaceutico I.N.M.I. Lazzaro Spallanzani I.R.C.C.S.
P	A	COMPONENTE	Dott. Giovanni REZZA	Direttore Centro Operativo AIDS Istituto Superiore di Sanità
P	A	COMPONENTE	Dott. Salvatore SQUARCIONE	Direttore Sanitario I.N.M.I. Lazzaro Spallanzani I.R.C.C.S.
P	A	COMPONENTE	Dott. Vittoradolfo TAMBONE	Esperto di Bioetica, Dipartimento di Antropologia ed Etica applicata, Libera Università Campus Bio-Medico

Ufficio di segreteria:

P	A	SEGRETARIO SCIENTIFICO	Dott. Enrico GIRARDI	Vice Direttore Scientifico I.N.M.I. Lazzaro Spallanzani I.R.C.C.S.
P	A	SEGRETARIO AMMINISTRATIVO	Dott.ssa Lorena FIORENTINI	Collaboratore Amministrativo presso la Direzione Scientifica I.N.M.I. Lazzaro Spallanzani I.R.C.C.S.
